# Quantifying Morphological Parameters of the Terminal Branching Units in a Mouse Lung by Phase Contrast Synchrotron Radiation Computed Tomography

**DOI:** 10.1371/journal.pone.0063552

**Published:** 2013-05-21

**Authors:** Jeongeun Hwang, Miju Kim, Seunghwan Kim, Jinwon Lee

**Affiliations:** 1 School of Interdisciplinary Bioscience and Bioengineering, POSTECH, Pohang, Republic of Korea; 2 Department of Physics, POSTECH, Pohang, Republic of Korea; 3 Institute for Edge of Theoretical Science, POSTECH, Pohang, Republic of Korea; 4 Department of Mechanical Engineering, POSTECH, Pohang, Republic of Korea; Pulmonary Research Institute at LungClinic Grosshansdorf, United States of America

## Abstract

An effective technique of phase contrast synchrotron radiation computed tomography was established for the quantitative analysis of the microstructures in the respiratory zone of a mouse lung. Heitzman’s method was adopted for the whole-lung sample preparation, and Canny’s edge detector was used for locating the air-tissue boundaries. This technique revealed detailed morphology of the respiratory zone components, including terminal bronchioles and alveolar sacs, with sufficiently high resolution of 1.74 µm isotropic voxel size. The technique enabled visual inspection of the respiratory zone components and comprehension of their relative positions in three dimensions. To check the method’s feasibility for quantitative imaging, morphological parameters such as diameter, surface area and volume were measured and analyzed for sixteen randomly selected terminal branching units, each consisting of a terminal bronchiole and a pair of succeeding alveolar sacs. The four types of asymmetry ratios concerning alveolar sac mouth diameter, alveolar sac surface area, and alveolar sac volume are measured. This is the first ever finding of the asymmetry ratio for the terminal bronchioles and alveolar sacs, and it is noteworthy that an appreciable degree of branching asymmetry was observed among the alveolar sacs at the terminal end of the airway tree, despite the number of samples was small yet. The series of efficient techniques developed and confirmed in this study, from sample preparation to quantification, is expected to contribute to a wider and exacter application of phase contrast synchrotron radiation computed tomography to a variety of studies.

## Introduction

The lung airway tree is a complex three-dimensional tube network based on dichotomous branching down to the terminal bronchioles and alveolar sacs. All the basic features of breathing, such as the highly heterogeneous distribution of ventilation, gas mixing between adjacent airways or acini, pendelluft and similar mixing phenomena, are the outcome of the morphology of the airway tree [1]–[17]. Although the morphology of the large airways has been extensively studied, the morphology of the small airways and terminal alveolar units (terminal bronchioles and alveolar sacs) have not been thoroughly investigated experimentally because of various technical limitations – such as insufficient imaging resolution. Because of the lack of understanding of the terminal units, the flow distribution in the airways could not be analyzed exactly, but rather has been estimated based on a simple models such as Murray’s law, which is based on an unrealistic (symmetric) geometry [9].

Furthermore, morphological information about the respiratory zone is needed to understand some pulmonary diseases. In pulmonary diseases such as chronic obstructive pulmonary disease [18]–[20], emphysema [21]–[22], asthma [23], and lung cancer [24], the disease progresses with a process called remodeling, which is a term that describes the destructive changes of the pulmonary microstructures [25]. Understanding the remodeling process is essential in the pathophysiology of pulmonary diseases [25], and any convenient means of monitoring the morphological parameter values for the respiratory zone can greatly aid early detection and treatment. However, conventional imaging techniques can yield only limited information about the respiratory zone.

Silicone casting is one of the most common methods to attain morphology data from the lung. A massive database summarizing the morphological parameters of the airways in various species was produced by silicone casting and manual measurements in millimeter scale resolutions [26]. Recently, a state-of-the-art silicone casting methodology was developed, combining micro-CT imaging with a 53 µm voxel size and a simulated annealing algorithm that computationally detects the airway bifurcations and measures morphological parameters from the CT images [27]–[28]. However, the resolution limited its application to the conducting airways.

In another imaging technique, the histological biopsy, lung tissue samples should be fixed, stained, embedded, and sliced so that they can be observed under optical microscopes [29]. The 3D information is inevitably lost during the process and decomposed into a 2D dimensionality. There exist sophisticated stereology methods to retrieve the 3D information from 2D images, but statistical prerequisites and yield estimations about the respiratory zone microstructures are required [30]–[31]. The stereology method works well for quantifying the mean free path or surface area per volume ratio, but other parameters such as the volume of an individual sac, mother-to-daughter diameter-reduction ratios, and asymmetry ratios at bifurcations are difficult to estimate from 2D images.

An ideal solution for imaging the respiratory zone is phase contrast synchrotron radiation computed tomography (PCSRCT). PCSRCT enables high resolution 3D imaging of the respiratory zone by utilizing the phase effect between air and tissue, thereby providing sharp images of the fine morphology of the terminal airways without using any contrast enhancement technique [32]–[33].

We developed a series of efficient techniques, including lung sample preparation, beam line settings, and an image processing scheme optimal for PCSRCT of the terminal branching units in the respiratory zone of a mouse lung. Using the methodology developed in this study, we successfully identified the connected 3D structure of the terminal alveolar units, and quantified morphological parameters. Although there have been reports on the three-dimensional morphology of respiratory zone microstructures [34]–[35] and individual alveoli [36]–[40], this is the first ever report regarding the connected 3D structure of the terminal alveolar units and the quantitative data of the asymmetry between alveolar sacs.

## Materials and Methods

### Lung Sample Preparation

An 8 week old male mouse of the C57BL/6 strain was used in this study, and all the procedures associated with the animal experiment were approved by the POSTECH Institutional Animal Care and Use Committee (approval ID: 2010-01-0015).

The lung sample was prepared following the instillation-based inflation-fixation method established by Heitzman [41]–[42]. The lung fixative subcommittee of the Society of Toxicologic Pathology strongly recommends the use of intratracheal instillation in sample preparation for quantitative studies on the morphometry of the alveoli [43]. Heitzman’s method has two advantages for morphology studies: 1) the microstructure of the air-tissue boundary is preserved [42], [44] and 2) the fixation stability of the inflation-fixed lung sample is superb, resulting in little or no shrinkage over time [44].

The mouse was humanely sacrificed by intraperitoneal administration of 2,2,2-tribromoethanol. Then the fixative, made of 50% (by volume) polyethylene glycol 400, 25% ethyl alcohol (95%), 10% formaldehyde (37%), and 15% double-distilled water was gently injected through the tracheal intubation, inflating the lung to the maximal volume *in situ*. The thorax was opened, and the whole lung was taken out of the thorax and immersed in the same fixative until the lung was completely de-gassed, which took 7 days. The fixed whole-lung sample was air dried with a blower for 72 hours in a fume hood. Air drying for 72 hours was enough to remove all the liquid from the sample, and the air lumen became air-filled after drying. The lung sample maintained its inflated state thereafter, keeping the airspace microstructures intact. The right lower lobe of the lung sample was separated and underwent PCSRCT one day after the preparation.

### PCSRCT

PCSRCT was performed at the 6D biomedical beam line in the Pohang Light Source (PLS). The technical specifications of the light source and the beam line attributes are described on the PLS website [45], and the principle of phase contrast tomography can be found elsewhere [46].

**Figure 1:**
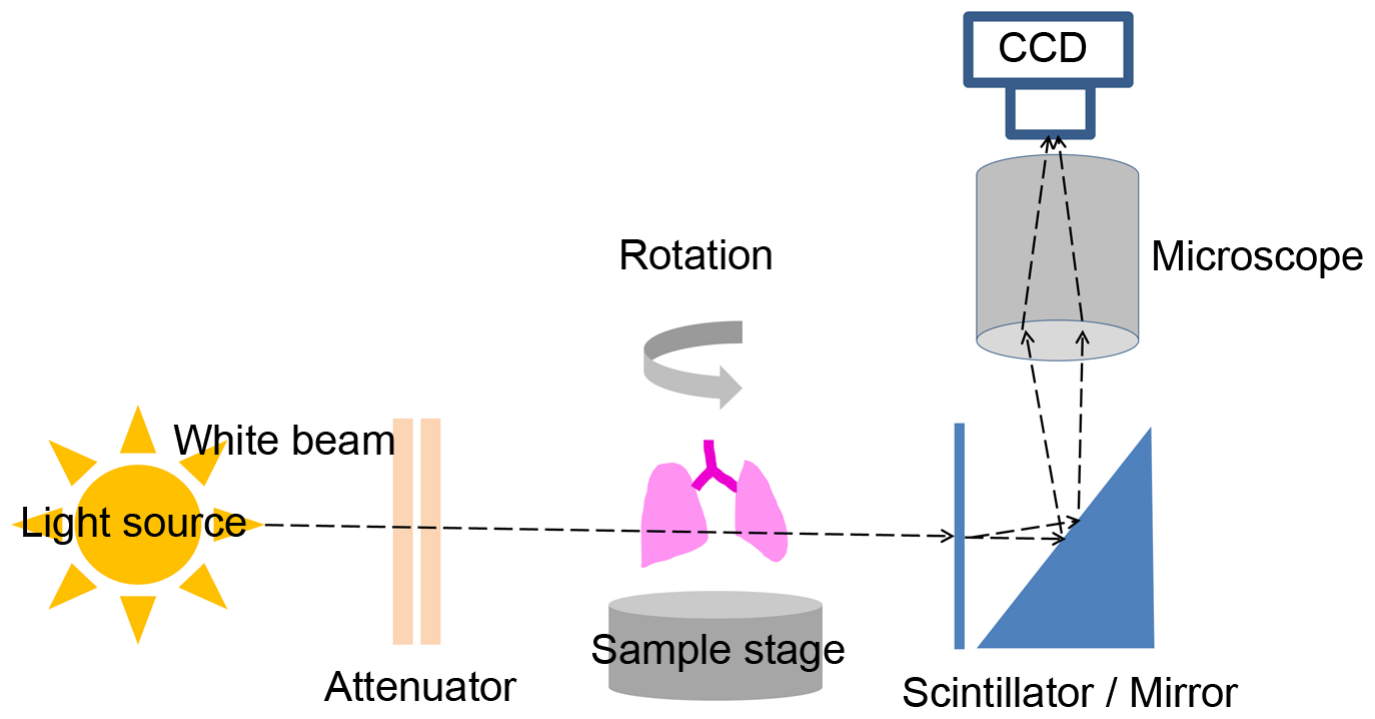
A schematic diagram of the beam line settings for PCSRCT imaging. PCSRCT was performed in PLS 6D biomedical beam line. The white beam was attenuated by a 1 mm silicon wafer before reaching the sample. The sample was rotated automatically on a sample stage, taking 500 projection images covering 180°. The beam propagated 18 cm from the sample to a CdWO_4_ scintillation crystal and was magnified 5X. All of the settings were fine -tuned for optimal image acquisition.

Figure 1 schematically shows the in-line settings, which were fine-tuned to achieve the best contrast. A silicon wafer of 1 mm thickness was used as the white beam attenuator, a CdWO_4_ crystal was used as the scintillator. We used the propagation based technique [32], and the sample-to- detector distance was 18 cm. The mean photon energy was 22 keV, and the bandwidth of the beam ΔE/E was 62%. While the sample stage rotated 180°, 500 projection images were taken using a 5× relay lens and a CCD camera with 4008×2672 pixels (VM-11M5-MF10 Vieworks Co. Ltd.). With these settings, one pixel corresponds to a scale length of 1.74 µm.

### Image Processing

**Figure 2:**
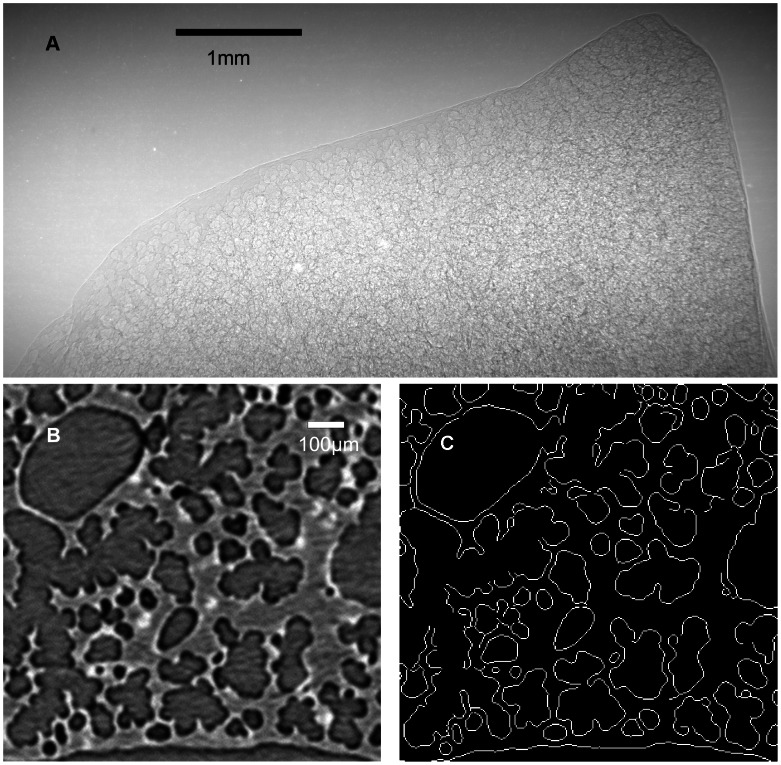
PCSRCT of the lung sample. (A) shows a projection image of a mouse lung sample. (B) is a part of a slice reconstructed from a projection image set. Air-tissue boundaries are clearly revealed. (C) shows computationally extracted gas exchange surface using an edge detection algorithm.

In a raw projection image (Figure 2A), information from all the spaces along the path of the synchrotron radiation is overlapped, so hardly anything local is discernible. To retrieve local information, image reconstruction is needed. Reconstruction yields cross-sectional images as shown in Figure 2B, and the cross-sectional images can be stacked to produce a 3D image. The filtered-back projection algorithm [47], embedded in the commercial software Octopus 8.5 (inCT, Belgium), was used in this study for reconstruction. The reconstruction process yielded a stack of 1600 contiguous slices, where each slice had 2000×2000 pixel size in 2-dimension. The thickness of each slices corresponded to 1.74 µm, and each pixel on a slice had scale of 1.74 µm×1.74 µm.

For clear visualization of the characteristic morphology and easy quantification of the morphometric parameters, image segmentation followed the reconstruction process, where the voxels in every image were classified into two different groups: air-tissue boundary voxels and non-boundary voxels. Threshold-based segmentation together with the region-growing algorithm is commonly used for this type of segmentation [39]–[40], but it often requires a substantial amount of time and manual workload, especially when applied to the phase contrast images of highly complicated geometries. Phase contrast imaging gives sharp edges on the air-tissue boundary, in such a way that a double layer with two different intensities, one very high and the other very low, is formed along the boundary. Voxels corresponding to both air space and tissue show medium intensity. This is a distinctive difference from the attenuation based CT images, and it is very easy to locate boundaries from the PCSRCT images.

Considering the basic feature of the PCSRCT images, Canny’s edge-detection algorithm [48] was used in this study instead of conventional segmentation methods for extracting the air-tissue boundaries. Segmentation based on edge detection fits well with the concept and the unique advantage of phase contrast imaging, which essentially enhances the phase edges. Matlab 7.8.0 was used for edge detection by creating binary outputs from the image dataset in such a way that the air-tissue boundaries were set to 1 and the other areas (both air lumen and tissue) were set to 0. Segmentation based on edge detection is very efficient because of its simplicity and reproducibility. Canny’s edge detector does not require high computation power, is very fast and is completely reproducible. In fact, it can yield results in seconds for a reconstructed image slice with 2000×2000 pixels on a moderate desktop PC.

No vessel or artery in our lung sample contained air; thus, all the air-tissue boundaries obtained by imaging in this study were exclusively the edges of the airspace lumen.

**Figure 3:**
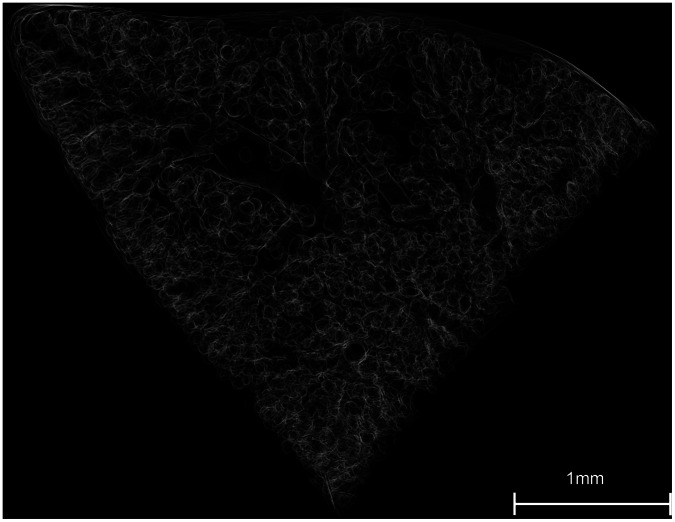
A 3D rendering of gas exchange surfaces. The figure shows a part of a mouse lung sample that was reconstructed, binarized, and rendered in 3D. The thickness of the presented sample part was 348 µm. Detailed morphology and relative positioning of the ducts, sacs, and larger airways are visualized.

The image stack was rendered into a 3D structure of connected airway lumens, as shown in Figure 3 and Video S1. Only a thin part of the whole dataset is visualized in Figure 3 and Video S1 for the ease of visual recognition. Sixteen terminal branching units were randomly selected from all over the dataset and identified for quantification. Amira 5.3.3 was used for the 3D rendering and the identification of the terminal units. The morphological parameters were analyzed quantitatively using Matlab 7.8.0 in combination with Amira 5.3.3.

### Quantifying Morphological Parameters

**Figure 4:**
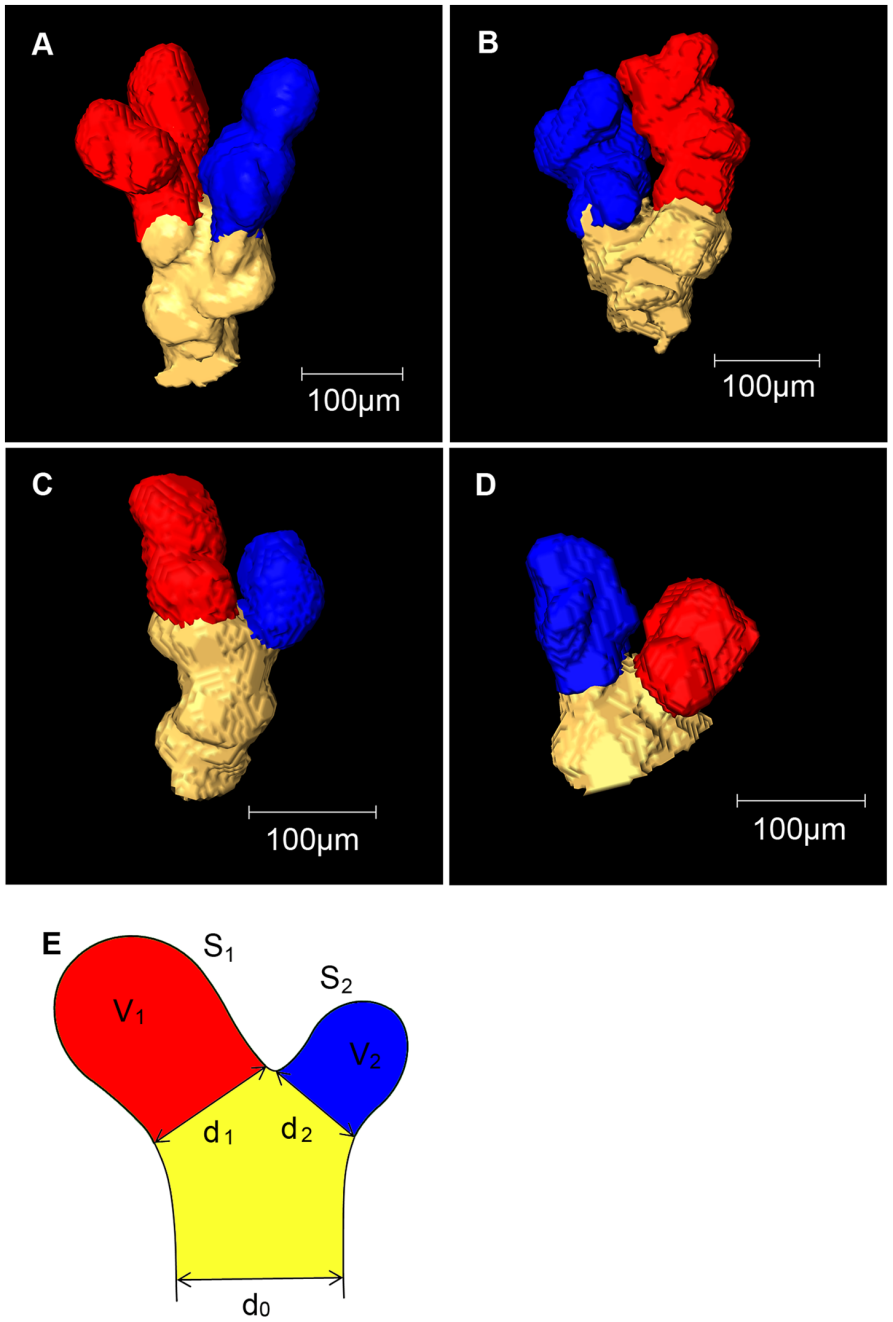
Isosurface visualization of the terminal branching units. A terminal branching unit is defined as a set of a “mother” terminal bronchiole and two sibling “daughter” alveolar sacs. Among the sixteen terminal branching units that had extracted randomly from the right lower lobe, the four units, unit #1, #2, #3, and #4 in Table 1 are depicted accordingly. (A) Unit #1, (B) Unit #2, (C) Unit #3, (D) Unit #4. The rotational movie of the terminal branching unit #1 was recorded and presented in Video S2. Designations of the morphological parameters d_0_, d_1_, d_2_, S_1_, S_2_, V_1_, and V_2_ are schematically shown in (E).

Each terminal branching unit consists of a “mother” terminal bronchiole and its two “daughter” alveolar sacs attached distal to the bifurcation point. Among the sixteen terminal branching units, unit #1, #2, #3, and #4 are visualized in Figure 4A, B, C, and D. Morphological parameters were measured as schematically described in Figure 4E and are described as follows: d_0_, the mother terminal bronchiole diameter; d_1_ and d_2_, the mouth diameter of the major and minor daughter alveolar sacs; S_1_ and S_2_, the surface areas of the major and minor daughter alveolar sacs; and V_1_ and V_2_, the volumes of the major and minor daughter alveolar sacs.

The mouth diameter of a daughter sac was defined as the boundary formed along the baselines of the sac at the bifurcation. To estimate the mouth diameter, for each mouth, lengths of a longer axis and a shorter axis were measured and their geometric mean was adopted as a diameter value.

The surface areas were measured by counting the number of voxel faces confronting with the “interior” of the alveolar sac on the 6-neighbor basis and multiplying it with the voxel face area, 1.74 µm×1.74 µm. Detailed scheme could be found in MATLAB Central website [49].

## Results

To reveal the 3D connective structure in the respiratory zone of a mouse lung and to quantify the morphological parameters, PCSRCT was performed with a whole-lung sample fixed at maximum inflation *in situ* and air dried ex-vivo. From 500 CT projection images of the lung sample (Figure 2A), cross-sectional images were reconstructed yielding contiguous slices, one of which is shown in Figure 2B. Edge-based segmentation was applied to these images to extract clear and sharp boundaries of the alveolar lumen (Figure 2C). 1.74 µm isotropic voxel size was attained, which is one of the highest resolutions among the PCSRCT imaging outcomes applied to the lung respiratory zone. Slightly better resolution of 1.4 µm^3^ per voxel was achieved in a study conducted in the Swiss Light Source, TOMCAT, using heavy metal staining, which can enhance the spatial resolution but also introduces the potential risk of artifacts [39]. Other examples are the 12 µm isotropic voxel size in intact mice lung by Parsons *et al.* [50] and the 13 µm voxel size in postmortem non-invasive tomography from the Shanghai Synchrotron Radiation Facility by Zhang *et al.* [40]. It is noteworthy that a competitively high resolution was attained without staining in this study. Details of the micro-structures were clearly revealed in a broad range of scales from bronchi to terminal bronchioles and alveolar sacs. The adoption of an efficient segmentation method, Canny’s edge detection algorithm, made it possible to extract the air-tissue boundaries very sharply with a single voxel thickness (Figure 2C).

The extracted air-tissue boundaries were rendered into a 3D structure for visual investigation. In Figure 3, bronchi, small airways, and alveolar sacs are easily recognizable, and the relative positioning of each component is also very comprehensible. The 3D rendered structure was also recorded as a rotation movie in Video S1.

For the quantification of the morphological parameters, sixteen terminal branching units were extracted from different parts of the lung sample. Four of the terminal branching units, unit #1, #2, #3, and #4 are shown in Figure 4. The rotational movie of the terminal branching unit #1 was recorded and presented in Video S2. The alveolar sacs may contain one or more alveoli. Morphological parameters were measured as schematically described in Figure 4E and are summarized in Table 1.

**Table 1:** Morphological parameters measured in the sixteen terminal branching units.

	Diameter (µm)			Surface area (×10^4^ µm^2^)		Volume (nL*)	
	d_0_	d_1_	d_2_	S_1_	S_2_	V_1_	V_2_
Unit #1	88.6	83.6	67.2	8.79	6.95	0.833	0.607
Unit #2	87.7	73.7	68.8	8.10	8.95	0.701	0.710
Unit #3	76.2	69.1	52.8	4.20	2.73	0.335	0.206
Unit #4	101	69.0	57.0	4.67	3.55	0.370	0.265
Unit #5	56.0	50.2	46.6	3.85	2.39	0.246	0.121
Unit #6	52.5	46.1	41.4	3.41	1.51	0.197	0.0634
Unit #7	60.4	45.0	29.0	1.62	0.851	0.0852	0.0305
Unit #8	123	92.0	64.8	7.52	4.51	0.646	0.396
Unit #9	102	80.0	63.0	5.24	5.17	0.489	0.437
Unit #10	73.3	77.2	61.9	4.11	3.07	0.319	0.202
Unit #11	97.0	75.0	73.6	3.77	4.67	0.340	0.432
Unit #12	80.1	70.0	62.3	3.23	3.25	0.217	0.242
Unit #13	86.1	83.0	60.0	9.77	4.12	0.920	0.308
Unit #14	98.0	73.8	64.5	7.75	3.90	0.680	0.284
Unit #15	70.3	55.7	42.7	4.35	3.34	0.364	0.279
Unit #16	67.6	52.2	44.8	2.01	3.43	0.115	0.205

* nano liter, 1 nL = 10^−9^ L = 10^6^ µm^3^.

Diameters, surface areas, and volumes were measured from the sixteen terminal branching units.

**Figure 5:**
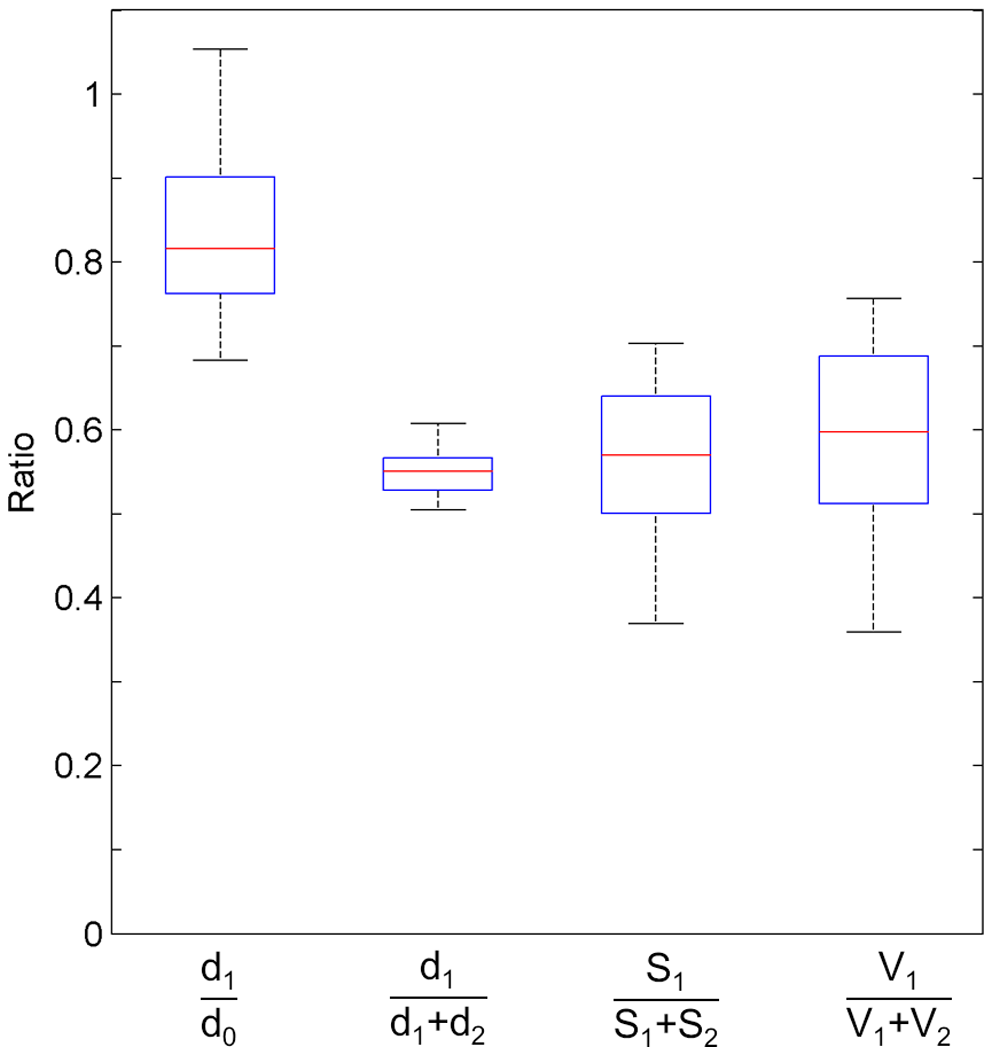
Various types of asymmetry ratios in sixteen terminal branching units of a mouse lung. Four types of asymmetry ratios, d_1_/d_0_, d_1_/(d_1_+d_2_), S_1_/(S_1_+S_2_), V_1_/(V_1_+V_2_) were measured from the sixteen terminal branching units. On each box, the red line is the median, the edges of the box are the 25th and 75th percentiles, and the whiskers extend to the most extreme data points. The median values are; d_1_/d_0_∶0.82, d_1_/(d_1_+d_2_): 0.55, S_1_/(S_1_+S_2_): 0.57, V_1_/(V_1_+V_2_): 0.60, respectively.

Shown in Figure 5 are various types of ratios of the morphological parameters given in Table 1, from which we can check the branching characteristics and/or asymmetries in the terminal units. This is the first ever attempt to measure the asymmetry ratios directly for the respiratory zone. The diameter reduction ratio, d_1_/d_0_ is one of the most fundamental morphological parameters of branching. d_1_/d_0_ ratios varied from 0.58 to 1.05 and the median was 0.82. Three normalized asymmetry ratios were defined as d_1_/(d_1_+d_2_), S_1_/(S_1_+S_2_), and V_1_/(V_1_+V_2_), each of which had median values of 0.55, 0.57, and 0.60 respectively.

## Discussion

In this study, an effective methodology for the quantitative assessment of 3D terminal branching structures was developed based on PCSRCT technology.

### Sample Preparation

A unique aspect of our methodology is that we introduced the Heitzman’s inflation-fixation method [41]–[42] to the sample preparation for the PCSRCT. The method was developed in the 1980s for micro-CT preparation, where the lung samples were fixed in an air-filled inflation state so that the micro-CT could detect the difference in X-ray absorbance between air and tissue. This method is relatively inexpensive and easy, and it has been demonstrated that the delicate microstructures of the lung samples are well preserved without any significant shrinkage or distortion [42], [44]. Vasilescu *et al.* proposed an optimized murine lung preparation technique that was suitable for detailed structural evaluation via micro-CT, where they adopted Heitzman’s fixative with some modification in the concentration of PEG 400. They preferred perfusion rather than instillation that was originally proposed by Heitzman. Compared to the perfusion-based methods, instillation-based methods have drawbacks when the inflation pressure is not properly controlled [43]–[44]. In this study, both over-inflation and under-inflation artifacts were totally avoided by keeping the inflation pressure constant at 30 cm H_2_O. No signs of alveolar rupture or disconnected alveolar sacs were observed in our images. Instillation-based inflation-fixation methods are recommended by the lung fixative subcommittee of the Society of Toxicologic Pathology, and credited for quantitative studies on the morphometry of the alveoli [43]. We proved the viability of Heitzman’s method for lung sample preparation for PCSRCT.

### PCSRCT

The purpose of imaging in this study was to enable quantitative assessments of the terminal branching units in the respiratory zone. To achieve this goal, the imaging technology needed to be tomographic and high resolution.

The morphometric features of the airways and acini have traditionally been studied using silicone lung cast models [26], and it is still a developing technique for quantitative assessments [27]–[28]. However, the method’s compatibility for scales under 10 µm has yet to be verified.

The advantages and drawbacks of other imaging technology, such as cryo-sectioning, magnetic resonance microscopy, optical coherence tomography, laser scanning confocal microscopy, and earlier micro-CT are summarized by Tsuda *et al.* [36], who also showed that synchrotron radiation tomography is the ideal solution for imaging the lung microstructure. According to their criteria, the PCSRCT technique that we used in this study is a synchrotron radiation X-ray tomographic microscopy because the transmitted images were first recorded on a scintillation crystal and magnified 5× by a light microscope before being recorded on a CCD. Zhang *et al.* discussed the advantages of phase contrast tomography compared with attenuation based tomography [40].

PLS is a third-generation synchrotron facility and has a biomedical imaging beam line 6D [45]. The beam line routinely reaches sub-micrometer spatial resolution, but the resolution in our study was set to 1.74 µm isotropic voxel size, as a compromise to get the required field of view. The in-line setting of our study is summarized in materials and methods. All the control parameters, especially the sample-to-scintillator distance were fine -tuned to yield the clearest contrast on the air-tissue boundaries. The importance of fine -tuning the sample-to-scintillator distance was discussed by Liu *et al.* [51]. The projection numbers were also carefully selected. In our study, 500 projections covered 180° of the sample rotation. We found that fewer projections hampered the resolution, while more projections resulted in elongated imaging time and caused heat damage concerns.

### Segmentation

Another unique aspect of this study is the adoption of Canny’s edge detector in the segmentation of the lung microstructure tomography. It extracted the connective structures of the respiratory zone components with clear visibility when rendered as shown in Figure 2. Canny’s edge detection algorithm was developed for general-purpose edge detection, and is widely accepted in the computer vision studies. It finds the intensity gradient in digital images, which corresponds to phase effects developed on the air-tissue boundaries in our study. Unlike other conventional segmentations that binarize voxels to either air or tissue, in our study, the voxels were binarized to either an air-tissue boundary or non-boundary, which included both air and tissue. As described in materials and methods, the use of Canny’s edge detector method is advantageous in terms of both speed and reproducibility. It has been confirmed through this study that segmentation based on edge-detection is highly recommendable for quantitative assessments of phase contrast tomography, especially when the difference in intensity between air space and tissue interior is not significant.

### Morphological Parameters

Figure 4E shows the definitions of the terminal branching units and their morphological parameters. We defined a terminal branching unit as a “mother” terminal bronchiole with two “daughter” alveolar sacs. Most of the surfaces in the terminal branching units were alveolated. The alveoli had very ambiguous borders, so separating out an alveoli or counting the numbers of alveoli on a sac was almost impossible. We found out that the ambiguity in the borderlines of alveoli was an innate feature, not an imaging artifact resulting from insufficient resolution. As presented in Figure 4, the bifurcation points and sac mouths were relatively apparent, so defining and separating the daughter alveolar sacs and the mother terminal bronchiole was unambiguous. Major and minor daughter sacs were determined based on their mouth diameters.

From the sixteen terminal branching units, we successfully measured the mother terminal bronchiole diameter d_0_, mouth diameters of the major and minor daughter alveolar sacs d_1_ and d_2_, and the surface area and volume of the major and minor daughter alveolar sacs S_1_, S_2_, V_1_, and V_2_, which are summarized in Table 1. Terminal bronchiole diameter ranged from 52.5 µm (unit #6) to 123 µm (unit #8), mouth diameter of alveolar sac from 29 µm (unit #7, minor) to 92 µm (unit #8, major), alveolar sac surface area from 8510 µm^2^ (unit #7, minor) to 97700 µm^2^ (unit #13, major), alveolar sac volume from 0.0305 nL (unit #7, minor) to 0.920 nL (unit #13, major). Parameswaran *et al*. estimated mean alveolar volume of normal mice as 0.12 nL [37] and it is well between the range of our alveolar sac volume measures, though an alveolar sac may contain one or more alveoli.

Based on the morphological parameters in Table 1, we calculated a diameter reduction ratio d_1_/d_0_, and three types of normalized asymmetry ratios as summarized in Figure 5: d_1_/(d_1_+d_2_), S_1_/(S_1_+S_2_), and V_1_/(V_1_+V_2_), each of which had median values of 0.82, 0.55, 0.57, and 0.60, respectively. It was observed from the measured morphological parameters that bifurcations were asymmetric in the most distal end of the airway tree. Also, the median values of the measured asymmetry ratios are largely in agreement with other published dataset [10], [26] on larger airways of other species. No previous studies showed clearly whether bifurcations in the respiratory zone were asymmetric. The asymmetry ratio in bifurcation is one of the dominant determinants of flow characteristics. The asymmetry ratio determines the heterogeneous distribution of ventilation and plays important roles in air mixing under laminar flow conditions [10] and in achieving uniform gas concentrations [16]–[17]. It is also expected that asymmetry allows the airway tree to reduce the average delivery time of fresh air [15]. This finding may facilitate the identification of a proper principle underlying the morphology or morphogenesis in the respiratory zone, which, unlike the conducting zone, is still not well established.

It is interesting to note that the diameter reduction ratios d_1_/d_0_ are more variable than the normalized sibling diameter ratios d_1_/(d_1_+d_2_), which may imply that the diameter of a duct is determined predominantly by the diameter of its sibling duct rather than that of its mother throughout morphogenesis.

The quantitative imaging scheme outlined in this study seems to be applicable to histopathological studies concerning airspace remodeling, such as quantifying the severity of airspace remodeling in diseased lungs. The morphological parameters data may also contribute to numerical simulations by making models more delicate and realistic, resulting in enhanced prediction capabilities.

## Supporting Information

Video S1 **A rotational movie of the 3D-rendered gas exchange surfaces.** A snapshot of this movie is presented in Figure 2. A small part of 348 µm thickness is shown for ease of visual inspection. Large airways, small airways, ducts, and alveolar sacs are visible, and the relative positions are easily appreciated.
(MPG)

Video S2 **A rotational movie of the terminal branching unit A.** A snapshot of this movie is shown in figure 4A. The detailed microstructures of a terminal branching unit are visible. The morphological parameters are in Tables 1.
(MPG)
